# Implication of the Association of Fibrinogen Citrullination and Osteoclastogenesis in Bone Destruction in Rheumatoid Arthritis

**DOI:** 10.3390/cells9122720

**Published:** 2020-12-20

**Authors:** Ji Soo Kim, Mikyung Choi, Ji Yong Choi, Joo Yeon Kim, Jeong Yeon Kim, Jin-Su Song, Lionel B. Ivashkiv, Eun Young Lee

**Affiliations:** 1 Department of Family Medicine, Seoul National University Hospital, Seoul 03080, Korea; clarekim89@gmail.com; 2 Department of Chemistry, Seoul National University, Seoul 08826, Korea; choi-mi-kyung@hanmail.net (M.C.); dew96@daum.net (J.-S.S.); 3 Division of Rheumatology, Department of Internal Medicine, Seoul National University College of Medicine, Seoul 03080, Korea; jy9793@gmail.com (J.Y.C.); jooyeon0106@gmail.com (J.Y.K.); jykim3607@naver.com (J.Y.K.); 4 Arthritis and Tissue Degeneration Program and David C. Rosensweig Center for Genomics Research, Hospital for Special Surgery, New York, NY 10021, USA

**Keywords:** citrullinated fibrinogen, osteoclastogenesis, rheumatoid arthritis

## Abstract

Immune complexes containing citrullinated fibrinogen are present in the sera and synovium of rheumatoid arthritis patients and potentially contribute to synovitis. However, fibrinogen can inhibit the osteoclastogenesis of precursor cells. We investigated the direct effect of citrullinated fibrinogen on osteoclastogenesis to understand the role of citrullination on bone erosion of rheumatoid arthritis patients. We evaluated the fibrinogen citrullination sites using mass spectrometry and quantified osteoclast-related protein and gene expression levels by Western blotting, microarray, and real-time polymerase chain reaction. Differences in spectral peaks were noted between fibrinogen and citrullinated fibrinogen at five sites in α-chains, two sites in β-chains, and one site in a γ-chain. Transcriptome changes induced by fibrinogen and citrullinated fibrinogen were identified and differentially expressed genes grouped into three distinctive modules. Fibrinogen was then citrullinated in vitro using peptidylarginine deiminase. When increasing doses of soluble fibrinogen and citrullinated fibrinogen were applied to human CD14+ monocytes, citrullination restored osteoclastogenesis-associated changes, including NF-ATc1 and ß3-integrin. Finally, citrullination rescued the number of osteoclasts by restoring fibrinogen-induced suppression of osteoclastogenesis. Taken together, the results indicate that the inhibitory function of fibrinogen on osteoclastogenesis is reversed by citrullination and suggest that citrullinated fibrinogen may contribute to erosive bone destruction in rheumatoid arthritis.

## 1. Introduction

Fibrinogen is a 340 kDa triglobular glycoprotein produced in the liver and comprising pairs of α, β, and γ chains linked by disulfide bonds [1,2,3]. Fibrinogen has a bifunctional domain structure and plays an important role not only in the blood coagulation system, but also in inflammation and tissue repair [3]. As part of the pro-inflammatory response, fibrinogen induces phagocyte recruitment via interaction with complement receptors (CD11b/CD18) and Toll-like receptor 4 (TLR4) [4]. A conformational change in fibrinogen has been shown to regulate the CD11b/CD18 receptor-binding site at the γ C-terminal end [5]. Based on a previous study, fibrinogen has an osteoprotective effect on bone and osteoclast precursor cells [6]. Specifically, fibrinogen binding to CD11b/CD18 results in strong inhibition of osteoclastogenesis induced by the receptor activator of nuclear factor kappa-Β ligand (RANKL).

The pathogenesis of rheumatoid arthritis (RA) involves chronic inflammation accompanied by bone erosion in synovial joints. The presence of anti-citrullinated protein/peptide antibodies (ACPAs) is associated with the severity and prognosis of RA, and both in vitro and in vivo studies have shown that ACPAs contribute to bone destruction [7,8]. The targets of these ACPAs include post-translationally modified proteins in which the amino acid arginine is deiminated by the enzyme peptidylarginine deiminase (PAD) to form citrulline [9]. The epitopes of various target proteins, such as fibrinogen, fibronectin, collagen, and enolase, are citrullinated within the synovium of RA patients [10,11]. Among the citrullinated proteins, fibrinogen is predominant and detected as a circulated antigen [12,13,14,15]. Several studies have described citrullination of human fibrinogen at multiple sites, mainly by human PAD isoforms (PAD2 and PAD4), but also by rabbit skeletal muscle PAD (PAD2) [3,16,17]. The classical role of fibrinogen as an autoantigen of ACPAs has been studied, but not its direct effect on bone. Based on our previous research of the negative effect of fibrinogen on osteoclast formation, we hypothesized that citrullinated fibrinogen can function as an arthritogenic protein in RA and, in contrast to fibrinogen, promote bone destruction.

## 2. Materials and Methods

### 2.1. Preparation of Human Synovial Fluid

Synovial fluid samples were obtained from patients with RA (*n* = 5) or osteoarthritis (OA, *n* = 5) at Seoul National University Hospital. All RA patients fulfilled the 2010 American College of Rheumatology/European League against Rheumatism classification criteria for RA [18]. The synovial fluid samples were diluted 1:3 in Tris-buffered saline and sonicated for analysis.

### 2.2. Mapping of Fibrinogen Citrullination

#### 2.2.1. Fibrinogen Separation

The fibrinogen and citrullinated fibrinogen solutions containing 0.1 M dithiothreitol (DTT) were centrifuged at 9000 rpm for 20 min using an Amicon Ultra device (Millipore, Billerica, MA, USA). We added 6 M urea and centrifuged the solutions again three times. We performed isoelectric focusing (IEF) using a 7-cm immobilized pH gradient (IPG) strip (pH 5–8, nonlinear gradient; Bio-Rad, Hercules, CA, USA) and a Protean IEF Cell (Bio-Rad). Fibrinogen (50 μg) was mixed with lysis buffer (6 M urea, 2 M thiourea, 50 mM DTT, 2% 3-[(3-cholamidopropyl)dimethylammonio]-1-propanesulfonate, 0.5% Triton X-100, 40 mM Tris, 0.2% Bio-lyte 3/10 ampholyte; Bio-Rad) to a total volume of 130 μL and loaded onto the IPG strip. After active rehydration (50 V, 15 h), proteins were focused at 10 kV for a total voltage of 60 kV. The IPG strip was placed in equilibration solution (6 M urea, 2% SDS, 0.375 M Tris-HCl, pH 8.8, 20% glycerol, 130 mM DTT) for 10 min and then transferred twice to another equilibration solution that did not contain DTT. Two-dimensional SDS-polyacrylamide gel electrophoresis was carried out with a 10% acrylamide gel using a Mini-Protean 3 Cell system (Bio-Rad) running a suitable buffer (25 mM Tris, 192 mM glycine, 0.1% SDS). The gel was stained with Coomassie Brilliant Blue R-250 (Bio-Rad).

#### 2.2.2. In-Gel Digestion

The spots excised from the gel were washed with 50 mM ammonium bicarbonate for 5 min, and then with 50 mM ammonium bicarbonate/100% acetonitrile (1:1) for 20 min. The liquid was discarded and washing repeated. The gel pieces were shrunk by dehydration in acetonitrile for 3 min, dried in a SpeedVac concentrator (Thermo Scientific, Rockford, IL, USA), swollen in 10 μL of 25 mM ammonium bicarbonate buffer containing 10 μg/mL trypsin (Sigma-Aldrich, St. Louis, MO, USA), and incubated overnight at 37 °C.

#### 2.2.3. In-Solution Digestion and Modification with Phenylglyoxal Monohydrate (PGM)

Fibrinogen was dissolved in deimination buffer (80 mM Tris-HCl, pH 7.6, 8 mM CaCl2, 4 mM DTT) to a concentration of 1 mg/mL. Citrullinated fibrinogen was digested by trypsin at 37 °C overnight, and we mixed 10 μL of the digested solution with 30 μL of trifluoroacetic acid (Sigma-Aldrich). Next, we added 10 μL of 50 mM PGM (Sigma-Aldrich) and left the mixture to act at 37 °C for 3 h. The reaction mixture was dried in a SpeedVac concentrator and resuspended in 10 μL of distilled water. Subsequently, we purified the modified peptides for mass spectrometric analysis using Ziptip (Millipore, Watford, UK).

#### 2.2.4. Matrix-Assisted Laser Desorption Ionization-Time of Flight (MALDI-TOF) Mass Spectrometry (MS)

We mixed 1 μL of the sample with 1 μL of matrix solution (10 mg of α-cyano-4-hydroxycinnamic acid, from Sigma-Aldrich; 0.1% trifluoroacetic acid in 1 mL of acetonitrile/distilled water, 7:3, *v*/*v*) and loaded 1 μL of the mixture onto the plate. After drying under vacuum, MALDI mass spectra were obtained using an Autoflex α MALDI-TOF mass spectrometer (Bruker Daltonics, Bremen, Germany).

### 2.3. In vitro Citrullination of Fibrinogen

To obtain citrullinated fibrinogen, bovine fibrinogen (Sigma-Aldrich) and human fibrinogen (Sigma-Aldrich) were each solubilized in phosphate-buffered saline (1 mg/mL) and citrullinated in vitro using either PAD2 (2U; Sigma-Aldrich) or human recombinant PAD4 (PAD4; 2U; ModiQuest Research, Nijmegen, The Netherlands). The reaction mixtures were incubated at 37 °C for 2 h [19].

### 2.4. Cell Culture and Osteoclast Differentiation

Peripheral blood mononuclear cells (PBMCs) were isolated from healthy donors and the buffy coat separated using Ficoll-Paque (GE Healthcare, Little Chalfont, United Kingdom). CD14+ monocytes were selected using beads, as recommended by the manufacturer (Miltenyi Biotec, Bergisch Gladbach, Germany). Monocytes were cultured with 20 ng/mL macrophage colony-stimulating factor (M-CSF; Sigma-Aldrich) for 1 day in Minimum Essential Medium Eagle—Alpha Modification (α-MEM; Gibco, BRL, Breda, the Netherlands). Monocyte-derived osteoclast precursor cells were plated in 96-well plates at a cell density of 1 × 10^6^ cells per well. The precursor cells were cultured for an additional 5 days, with cytokines M-CSF and RANKL (40 ng/mL; Peprotech, Rocky Hill, NJ, USA) being replenished every other day. M-CSF and RANKL were supplemented in the control cells. On day 6, cells were fixed and stained for the tartrate-resistant acid phosphatase (TRAP) assay as recommended by the manufacturer (TRAP kit, Sigma-Aldrich).

### 2.5. Western Blot Analysis

On day 7, protein was extracted from cells using Mammalian Protein Extraction Reagent (Thermo Scientific) according to the manufacturer’s instructions. Protein samples were resolved using the iBlot 2 Dry Blotting System (Life Technologies, Carlsbad, CA, USA). Membranes were treated with the following primary antibodies: anti-NF-ATc1 (Santa Cruz Biotechnology, Santa Cruz, CA, USA), anti-β3-integrin (Cell Signaling Technology, Danvers, MA, USA), anti-GAPDH (Santa Cruz Biotechnology), and anti-citrullinated fibrinogen (ModiQuest Research). The membranes were then treated with the corresponding secondary antibodies. Detection was performed using the Luminata Forte Western HRP Substrate (Millipore).

### 2.6. Immunoprecipitation

For protein sample preparation, RA (*n* = 2) and OA (*n* = 2) synovial fluids were digested with 2 μg/mL hyaluronidase (Sigma-Aldrich) and centrifuged at 15,000 rpm for 15 min at 4 °C. Anti-fibrinogen antibody (Abcam, Cambridge, UK) was added to synovial fluid samples and incubated overnight at 4 °C. They were immunoprecipitated over 2 h at 4 °C with protein G (Roche, Basel, Switzerland) and centrifugated. SDS-PAGE sample buffer was added to the beads and heated for 10 min at 95 °C. Then, the supernatants were collected after centrifugation and immunoblotted with anti-modified citrulline antibody (Sigma-Aldrich).

### 2.7. Bone Resorption Assay

Bone resorption activity was determined using the OsteoLyse assay kit (Lonza, Allendale, NJ, USA) according to the manufacturer’s protocol. Briefly, human CD14+ monocytes were cultured on a calcium phosphate-coated 96-well plate (5 × 10^4^ cells/well) in the presence of M-CSF (30 ng/mL) and soluble RANKL (100 ng/mL) with different concentrations of human fibrinogen (Sigma-Aldrich) or citrullinated human fibrinogen (Cayman Chemical, Ann Arbor, MI, USA) for 6 days. The wells of the bone plates were rinsed with water and then incubated for 5 min at room temperature with 5% sodium hypochlorite. Wells were then rinsed with water, and cells were removed by mechanical agitation. Microscopy images were then acquired using a Leica (DM2500) and pit area was calculated using the Metamorph image-analysis software.

### 2.8. Gene Expression Analysis

#### 2.8.1. Real-Time Polymerase Chain Reaction (PCR)

On day 4, cells were collected and RNA extracted using the RNeasy Mini Kit (Qiazen, Hilden, Germany). Complementary DNA was obtained using the GoScript Reverse Transcription System (Promega, Fitchburg, WI, USA). The TaqMan Gene Expression Assay (Thermo Scientific) was used to quantify the expression of the NF-ATc1 (NFATC1), β3-integrin (ITGB3), TRAP (TRAP), cathepsin K (CTSK), osteoclast-associated receptor (OSCAR), and calcitonin receptor (CALCR) genes.

#### 2.8.2. Microarray

CD14+ monocytes were treated with M-CSF in the presence of fibrinogen or citrullinated fibrinogen. After 24 h, the total RNA in each group was amplified and purified using an Ambion Illumina RNA amplification kit (Ambion, Austin, TX, USA) to yield biotinylated cRNA according to the manufacturer’s instructions. Arrays were scanned using an Illumina bead array reader confocal scanner according to the manufacturer’s instructions. The quality of the hybridization and overall chip performance were monitored by visual inspection of both internal quality control checks and the raw scanned data. Raw data were extracted by the software provided by the manufacturer (Illumina GenomeStudio v2009.2 (Gene Expression Module v1.5.4), San Diego, CA, USA). For the analysis of transcriptional regulation, we used the web-based software TRRUST ver 2 for gene set enrichment analysis (GSEA) and the TRRUST database [20]. GSEA 4.0.3 software was used to perform the GSEA with a ranked list of genes originating from a previously reported osteoclast-specific gene set [21].

### 2.9. Statistical Analysis

Statistical analyses were performed using SPSS Statistics software V. 22.0 (IBM, Armonk, NY, USA). We obtained means and standard deviations. In the osteoclastogenesis study, group means were compared using the Kruskal–Wallis test. The Wilcoxon’s signed-rank test was used to determine significant differences between fibrinogen and citrullinated fibrinogen in osteoclast gene expression.

## 3. Results

### 3.1. Mapping of Fibrinogen Citrullination Sites in the Presence and Absence of PAD

**Figure 1:**
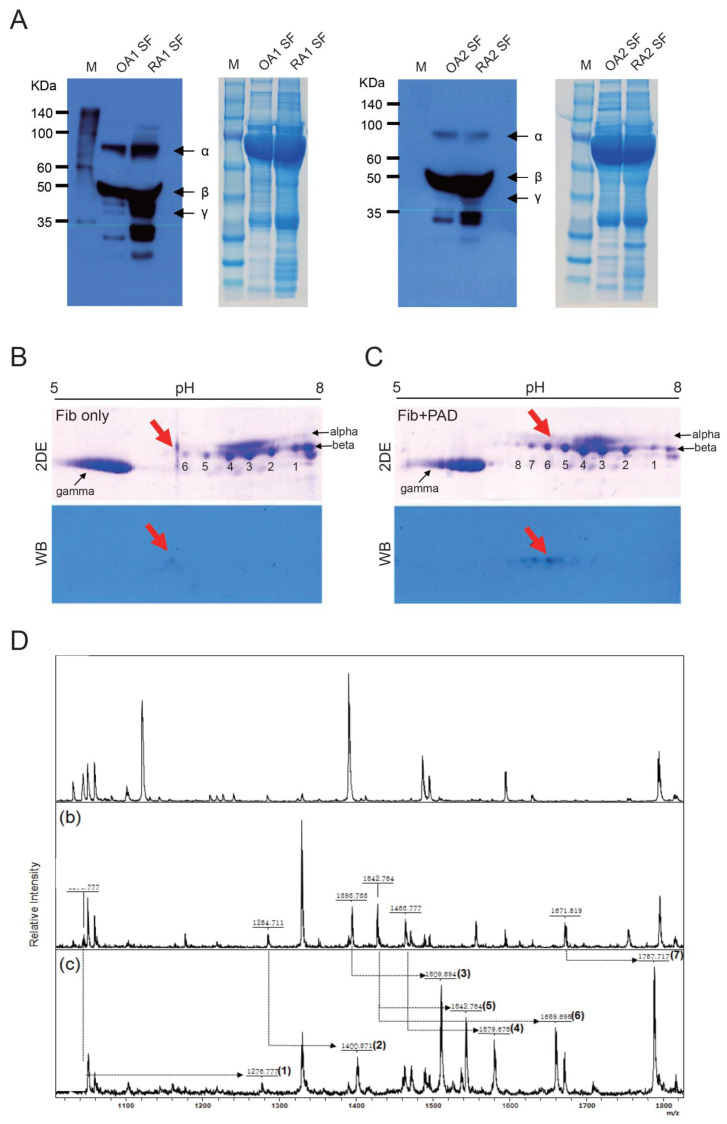
Citrullination of fibrinogen at α, β, and γ chains in the presence of peptidylarginine deiminase (PAD). (**A**) Level of citrullinated fibrinogen in synovial fluid (SF) from two osteoarthritis (OA SF1, OA SF2) and two rheumatoid arthritis (RA SF1, RA SF2) patients was detected by immunoprecipitation (IP) with anti-fibrinogen antibody and Western blot (WB) using anti-modified citrulline antibody. Coomassie, corresponding to each WB, of total protein in synovial fluid. Arrows indicate corresponding bands for α, β, and γ chains of fibrinogen. Fibrinogen from RA SF was more citrullinated than OA SF. (**B,C**) Spots for the α, β, and γ chains shifted in two-dimensional mapping of fibrinogen in the absence (B, upper row) or presence (C, upper row) of PAD2 (PAD). Shifts due to citrullination were confirmed by WB using anti-citrullinated fibrinogen antibody (lower rows of B and C). Arrows indicate corresponding spots between two-dimensional electrophoresis (2DE) and WB at a designated pH. (**D**) Citrullination sites (five in α, two in β, and one in γ chains) were detected by mass spectrometry via modification with phenylglyoxal monohydrate (PGM). Top panel = bovine fibrinogen (Fib.), Middle panel = Fib. + PAD, Bottom panel = Fib. + PAD + PGM.

We compared the extent of citrullination of synovial fluid fibrinogen in patients with active seropositive RA and OA. As predicted, a higher amount of citrullinated fibrinogen was observed in RA than in OA synovial fluid (Figure 1A).

Previous studies have described citrullinated sites on fibrinogen and α-enolase. We used two-dimensional SDS-PAGE and MALDI-TOF MS to detect the sites and extent of citrullination on fibrinogen. Modification of arginine residues to citrulline caused a pI change in SDS-PAGE and shifted spots on the electrophoresis gel to a lower pH. Compared to the spots obtained in the absence of PAD2, the spots obtained from PAD2-treated fibrinogen were shifted toward a lower pH for all three chain types (α, β, and γ; Figure 1B,C upper rows) [22]. To identify citrullination sites, spots of the relevant chain were cut and processed through gel digestion. Notably, PAD2-treated fibrinogen had darker spots in positions ‘5′ and ‘6′, whereas two spots newly appeared at a lower pH in positions ‘7′ and ‘8′. Corresponding changes were observed by Western blotting (Figure 1B,C lower rows). The successive decrease in spot intensity was indicative of successive citrullination of different arginine residues.

Spots that shifted after the addition of PAD2 to fibrinogen were evaluated by MS (Figure 1D). Because the peptide bond after citrulline cannot be cleaved by trypsin, mass peaks resulting from citrullination cannot be distinguished from those caused by miscleavage [23]. However, by modifying citrulline with PGM under acidic conditions, arginine residues that have been citrullinated can be identified based on a 116-Da increase in mass [24]. Using this approach, five sites were identified in α-chains, two sites in β-chains, and one site in a γ-chain (Table 1). Both arginine residues in the β-chains were citrullinated by PAD2, though only one of the two sites was detected in some experiments. When similar tests were run with PAD4 instead of PAD2, seven citrullinated sites were detected, six of which were the same as those resulting from the addition of PAD2.

### 3.2. Citrullinated Fibrinogen and Its Effect on the Gene Expression Profile of Human CD14+ Monocytes

**Figure 2:**
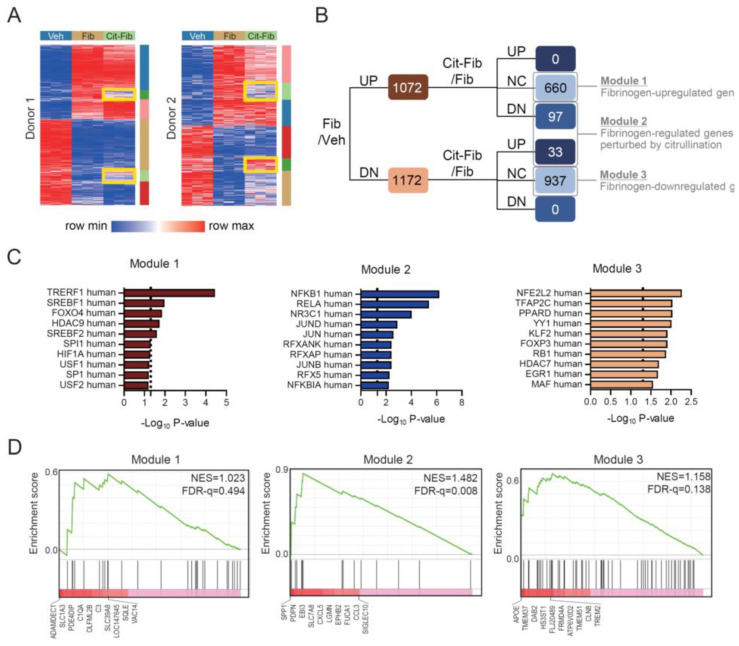
Differentially expressed genes (DEGs) in CD14+ sorted monocytes from two peripheral blood donors in the presence of fibrinogen or citrullinated fibrinogen. (**A**) Heatmap of DEGs. Veh = vehicle, Fib = bovine fibrinogen, Cit-Fib = bovine fibrinogen citrullinated by PAD2. Six different clusters of DEGs were presented by K-means clustering analysis. Yellow boxes indicate the clusters associated with restoration of fibrinogen-induced change by citrullination. (**B**) Fibrinogen-regulated genes perturbed by citrullination correspond to DEGs in the yellow box in (**A**). UP = upregulated genes, NC = no change in expression, DN = downregulated genes. (**C**) Gene set enrichment analysis of the module 1, 2, or 3 using the human TRRUST transcription factor database. Dashed line = *p*-value 0.05. (**D**) Gene set enrichment analysis of the module 1, 2, or 3 using osteoclast-specific gene set originated from ref. 21. NES = normalized enrichment score, FDR-q = q-value of false discovery rate.

We compared transcriptome changes induced by vehicle, fibrinogen, and citrullinated fibrinogen treatments on CD14+ sorted monocytes between two healthy human donors. First, we found 4127 genes significantly changed by fibrinogen compared to vehicle. This is shown as a heatmap including a group treated with citrullinated fibrinogen for each donor (Figure 2A). Using K-means clustering analysis, we found four distinguished gene sets among the fibrinogen-upregulated or -downregulated genes in each donor that had reversed patterns of gene expression with citrullinated fibrinogen (Figure 2A, yellow boxes). Based on the genes with a concordant pattern of expression between donors 1 and 2, we classified 1072 genes as significantly upregulated and 1172 genes as significantly downregulated (Figure 2B). Fibrinogen-upregulated and -downregulated genes were further classified by three modules according to the responsiveness to citrullination. Module 1 (*n* = 660) included the genes upregulated by fibrinogen and not affected by citrullination; module 2 (*n* = 130) included the genes upregulated or downregulated by fibrinogen but reversed significantly by citrullination; and module 3 (*n* = 937) included the genes downregulated by fibrinogen and not affected by citrullination. No gene significantly changed by fibrinogen was concordantly up- or downregulated by citrullination. Therefore, module 2 was the gene set with expression that was reactive to fibrinogen but perturbed by citrullination.

Next, we performed GSEA of the three modules for transcription factor-associated pathways. For module 1, the pathways associated with TREF1, SREBF1/2, FOXO4, and HDAC9 were significantly enriched (Figure 2C, left). Intriguingly, module 2 was significantly enriched in NFKB-related pathways (NFKB1, NFKBIA) and JUN-family member-associated pathways (JUN, JUNB, JUND; Figure 2C, middle). Module 3 was enriched in the pathways associated with transcription factors, including NFE2L2, TFAP2C, and PPARD (Figure 2C, right). We also performed GSEA using the gene list originating from differentially expressed genes between osteoclasts and CD14+ monocytes [21]; only module 2 was significantly enriched (Figure 2D). Collectively, the results indicate that fibrinogen-regulated genes perturbed by citrullination were associated with NFKB pathways and may contribute to osteoclastogenesis.

### 3.3. Citrullination Affects Fibrinogen-Induced Changes in Osteoclastogenesis-Related Gene Expression

**Figure 3:**
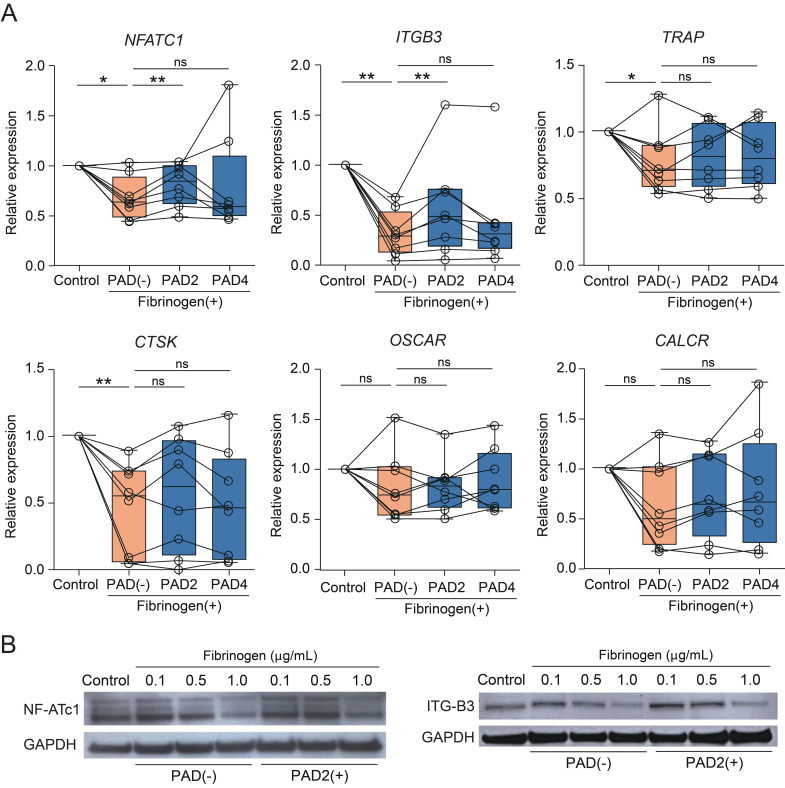
Gene and protein expression in osteoclasts in the presence of PAD. (**A**) Real-time PCR of cells cultured in the absence or presence of PAD2 or PAD4 at a dose of 1.0 μg/mL (*n* = 8). * *p*  <  0.05; ** *p*  <  0.01; ns—not significant. Individual values are plotted, and error bars represent SD. (**B**) Western blot of NF-ATc1, β3-integrin, and GAPDH. M-CSF and RANKL were supplemented in the control group.

To confirm the microarray findings from Figure 2D, we performed real-time PCR to quantify the mRNA expression of six canonical osteoclast marker genes (Figure 3A). PAD2 reaction with fibrinogen resulted in a significant increase in the expression of NFATC1 and ITGB3. Expression of TRAP, CTSK, OSCAR, and CALCR also tended to increase with the addition of PAD2 or PAD4, but the difference was not significant. Evaluation of NF-ATc1 and β3-integrin levels by Western blotting showed that PAD2 induced reversion of the fibrinogen-induced dose-dependent decrease (Figure 3B).

### 3.4. Citrullinated Fibrinogen Reverses the Inhibition of Osteoclastogenesis

**Figure 4:**
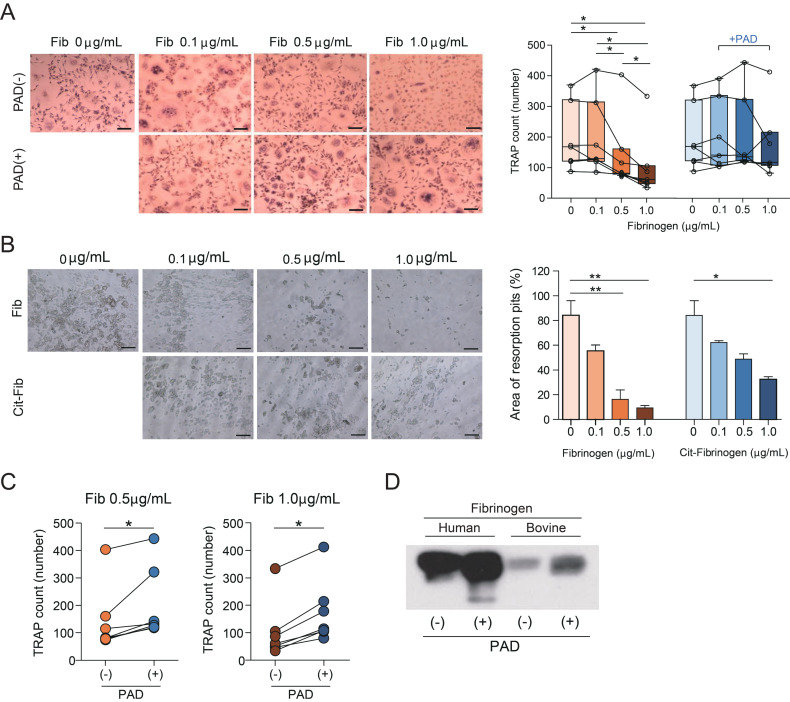
Reversion of dose-dependent inhibition of osteoclastogenesis in the presence of PAD. (**A**) Left: monocytes were cultured in the absence or presence of PAD2 (PAD) and stained for TRAP on day 6 (*n* = 6). M-CSF (20 ng/mL) and RANKL (40 ng/mL) were replenished every 3 days. M-CSF and RANKL were supplemented in the control group. Fib = bovine fibrinogen. Scale bar 200 μm. Right: number of TRAP-positive osteoclasts in the absence or presence of PAD. * *p*  <  0.05. Individual values are plotted, and error bars represent SD. (**B**) Left: bone resorption activity in the presence of fibrinogen or citrullinated fibrinogen for 6 days. Fib = human fibrinogen, Cit-Fib = citrullinated human fibrinogen. Scale bar 100 μm. Right: area of resorption pits (%). * *p*  <  0.05; ** *p*  <  0.01. (**C**) Number of TRAP-positive osteoclasts according to fibrinogen dose. * *p*  <  0.05. Individual values are plotted. (**D**) Western blot using an antibody against citrullinated fibrinogen and protein from reaction mixtures containing PAD with human fibrinogen or bovine fibrinogen incubated at 37 °C for 2 h.

Previous studies have shown that fibrinogen inhibits osteoclastogenesis in a dose-dependent manner [6]. To confirm this, CD14+ monocytes were supplemented with fibrinogen and the cells stained for TRAP on day 6 of culture (Figure 4A, upper row). To observe the effect of citrullinated fibrinogen on osteoclast formation, fibrinogen was reacted with PAD2 prior to being added to the monocytes. Though the number of osteoclasts significantly decreased with increasing fibrinogen dose (in the absence of PAD2), osteoclastogenesis was less inhibited when citrullinated fibrinogen was applied (Figure 4A). Similarly, citrullinated fibrinogen hindered the resorptive capacity of osteoclasts less (Figure 4B). We observed a significant difference in the number of osteoclasts between the two groups (i.e., osteoclastogenesis in the absence and presence of PAD2) at fibrinogen doses 0.5 μg/mL and 1.0 μg/mL (Figure 4C). Doses higher than 1.0 μg/mL inhibited osteoclast formation in both groups (data not shown), which may be attributable to factors other than PAD.

Previous studies have shown that either PAD2 or PAD4 was effective in citrullinating fibrinogen [17,25]. We tested the efficacy of PAD2 and level of citrullination of fibrinogen by reacting PAD2 with both human and bovine fibrinogen. Fibrinogen in its natural form is already citrullinated to a certain extent, which is greater in human blood than in bovine blood (Figure 4D). PAD2 modified fibrinogen to the citrullinated form regardless of whether fibrinogen was of human or bovine origin.

## 4. Discussion

RA is characterized by progressive destruction of articular cartilage and bone erosion. Fibrinogen is abundant in RA synovial fluid, but deiminated fibrinogen in synovial fluid is not specific for RA, as it is also detected in psoriatic arthritis, ankylosing spondylitis, and osteoarthritis [11]. Fibrinogen has been described as having an “anti-osteoclastogenic effect” [6,10]. In this study, we demonstrated that fibrinogen has an inhibitory effect on osteoclastogenesis, but its citrullinated form loses this osteoprotective property.

Previous studies on bone loss in RA have focused on fibrinogen as an autoantigen of the ACPA immune response [26]. Osteoclastogenesis and bone resorption have been induced by autoantibodies against citrullinated vimentin [27], and ACPAs have been shown to enhance osteoclast differentiation through PAD-dependent interleukin-8 [28]. However, a time-resolved kinetics study showed that osteoclast activation is inhibited by the early addition of a PAD inhibitor, even in the absence of ACPAs. As such, citrullinated fibrinogen may affect osteoclasts and precursor cells in an ACPA-independent manner.

Citrullinated fibrinogen could alter the inhibitory effect of fibrinogen on osteoclastogenesis. In an in vitro model of bone loss, increased citrullinated fibrinogen levels inhibited osteoclast formation less in a dose-dependent manner, which contrasts the effect of one or more immunogenic epitopes that may generate autoantibodies specific to RA. Citrullination of fibrinogen precedes osteoclast differentiation of the monocyte lineage, and the functional changes relative to bone are reflected in osteoclast-specific gene and protein expression. Citrullinated fibrinogen is more abundant in synovial fluid from RA patients than the synovial fluid from OA patients. Furthermore, in its natural form, fibrinogen of human and bovine origin is already citrullinated to a certain extent. The addition of PAD leads to increased production of citrullinated fibrinogen, further suggesting RA-specific elevation of the levels in synovial fluid. The increased amount of citrullinated fibrinogen detected in RA synovial fluid may be attributable to the severity or characteristic bone damage of RA. Therefore, abundant citrullinated fibrinogen is presumed to cause bone resorption in a RANKL-dependent pathological condition specific to RA [29,30].

Both types of PAD used in this study had similar sites of modification. Bovine fibrinogen has 71% sequence homology with human fibrinogen. The sequences adjacent to the newly citrullinated arginine in bovine fibrinogen are highly homologous to that of human fibrinogen (Figure S1). Thus, it is highly probable that the citrullination sites identified in bovine fibrinogen are also targets of citrullination in human fibrinogen. When we tested the peptides of citrullinated fibrinogen obtained from MS (Table 1) separately to evaluate the effect of each identified citrullination site, we observed no significant differences in osteoclast formation (data not shown). These findings suggest that the effect of citrullinated fibrinogen on osteoclast formation may be due to citrullination of sites important for receptor binding. Sokolove et al. demonstrated that citrullinated fibrinogen stimulates macrophages and enhances tumor necrosis factor (TNF) production via TLR4 and Myd88 [31]. A more recent study observed another pro-inflammatory effect of citrullinated fibrinogen; in addition to the classical way of triggering autoimmunity, citrullinated fibrinogen impaired the immunomodulatory function of bone marrow mesenchymal stem cells by triggering TLR [32]. Fibrinogen has also been linked to joint disease through the integrin αMβ2 binding motif [33]. As such, further study is needed to elucidate the mechanism of fibrinogen-receptor binding.

This study has several limitations. First, in vitro citrullination and osteoclast differentiation may not accurately reflect the physiological processes that occur in vivo. Second, we were not able to elucidate the nature of fibrinogen-receptor binding or whether fibrinogen within the synovial fluid acts in peptide form. The effect of citrullination on the function of fibrinogen in osteoclastogenesis may be the result of different binding affinities between fibrinogen and CD11b/CD18, or of fibrinogen binding to an array of receptors that produce a net effect of bone resorption. Finally, the effect of other citrullinated proteins, such as vimentin or enolase, on bone was not observed.

The role of citrullinated proteins in the pathophysiology of RA is not fully understood [27], but we provide another perspective of the role of fibrinogen on bone destruction within the framework of immunological activation and inflammatory pathways characteristic of RA, stressing the importance of further study of this perspective. Taken together, our data suggest that the function of fibrinogen is altered in the presence of PAD, and citrullination results in a loss of the osteoprotective effect of fibrinogen.

## Figures and Tables

**Table 1:** Citrullinated peptide sequences in the fibrinogen α, β, and γ chains.

	*m*/*z* Ratio	Residue Number	Peptide Sequence	MH+	Comparison to PAD4
**α-chain**					
(1)	1276.77	139-146	S**R**IEIL**R**R	1042.65	S**R**IEIL**R**R
(2)	1400.87	91-100	L**R**DSLFNYQK	1283.67	L**R**DSLFNYQK
(3)	1509.69	111-122	NIVELM**R**GDFAK	1392.73	
(4)	1579.67	229-241	MSTITGPVP**R**EFK	1462.77	MSTITGPVP**R**EFK
**β-chain**					
(5)	1542.76	16-28	VGLGA**R**GH**R**PYDK	1425.77	VGLGA**R**GH**R**PYDK
(6)	1659.69	16-28	VGLGA**R**GH**R**PYDK	1425.77	VGLGA**R**GH**R**PYDK
					KEEAPSL**R**PVPPPISGGGYR
**γ-chain**					
(7)	1787.71	169-183	IHDVTG**R**DCQDVANK	1670.79	-

R = citrulline (site citrullinated by PAD2), *m*/*z* ratio = mass-to-charge ratio, MH+ = positive ion mode.

## Supplementary Materials

The following are available online at https://www.mdpi.com/2073-4409/9/12/2720/s1, Figure S1: Sequence comparison between bovine and human fibrinogen according to α, β, and γ chains.
