# Supplementary material for: Implication of the Association of Fibrinogen Citrullination and Osteoclastogenesis in Bone Destruction in Rheumatoid Arthritis
Source: Cells. 2020 Dec 20;9(12):2720. doi: 10.3390/cells9122720 (PMC7766778; doi:10.3390/cells9122720)
Supplement: Supplementary file 1 [file cells-09-02720-s001.pdf]

Alpha chain

```
             81          91          101         111         121
Bovine       DQDFT SRINK LRDSL FNYQK NSKDS NTLTK NIVEL MRGDF AKANN NDNTF
Human          NQDFT NRINK LKNSL FEYQK NNKDS HSLTT NIMEI LRGDF SSANN RDNTY
             78          88          98          108         118

             131         141         151         161         171
Bovine       KQISE DLRSR IEILR RKVIE QVQRI KVLQK NVRDQ LVDMK RLEVD IDIKI
Human          NRVSE DLRSR IEVLK RKVIE KVQHI QLLQK NVRAQ LVDMK RLEVD IDIKI
             128         138         148         158         168

             181         191         201         211         221
Bovine       RSCKG SCSRA LEHKV DLEDY KNQQK QLEQV IAINL LPSRD IQYLP LIKMS
Human          RSCRG SCSRA LAREV DLKDY EDQQK QLEQV IAKDL LPSRD RQHLP LIKMK
             178         188         198         208         218

             231         241
Bovine       TITGP VPREF KSQLQ EAPLE
Human          PVPDL VPGNF KSQLQ KVPPE
             228         238
```

Beta chain

```
             1           11          21          31          41
Bovine       QFPTD YDEGQ DDRPK VGLGA RGHRP YDKKK EEAPS LRPVP PPISG GGYRA
Human          CVFLV KSQGV NDNEE GFFSA RGHRP LDKKR EEAPS LRPAP PPISG GGYRA
             24          34          44          54          64
```

Gamma chain

	161	171	181		191		201				
Bovine	KVVQL EANCQ	EPCQD	TVKIH	DVTG**R**	DCQDV		ANKGA	KESGL	YFIRP	LKAKQ	
Human		KVAQL	EAQCQ	EPCKD	TVQIH	DITGK	DCQDI	ANKGA	KQSGL	YFIKP	LKANQ
	163	173	183		193		203				

Figure S1. Sequence comparison between bovine and human fibrinogen according to α, β, and γ chains. **R** = citrulline (site citrullinated by PAD2 or PAD4), grey box = homologous sequence.
